# Toxicity Studies of *Lactobacillus plantarum* PS128^TM^ Isolated from Spontaneously Fermented Mustard Greens

**DOI:** 10.3390/foods8120668

**Published:** 2019-12-11

**Authors:** Po-Lin Liao, Chien-Chen Wu, Tai-Ying Chen, Ying-Chieh Tsai, Wu-Shun Peng, Deng-Jye Yang, Jaw-Jou Kang

**Affiliations:** 1 Institute of Food Safety and Health Risk Assessment, National Yang-Ming University, Taipei 112, Taiwan; smallfu0508@gm.ym.edu.tw (T.-Y.C.); djyang@ym.edu.tw (D.-J.Y.); 2 Institute of Biochemistry and Molecular Biology, National Yang-Ming University, Taipei 112, Taiwan; allen@benedbiomed.com (C.-C.W.); tsaiyc@ym.edu.tw (Y.-C.T.); 3 Microbiome Research Center, National Yang-Ming University, Taipei 112, Taiwan; catpet1022@hotmail.com

**Keywords:** 28 day repeated oral dose toxicity, genotoxicity test, *Lactobacillus plantarum* PS128^TM^, no-observed-adverse-effect-level (NOAEL)

## Abstract

Probiotics are extensively available to consumers; however, the use of probiotics may not always be safe, and there are few reports on their side effects, including those of *Lactobacillus*. *Lactobacillus plantarum* strain PS128^TM^ isolated from spontaneously fermented mustard greens in Taiwan was recently reported to exhibit probiotic properties. In this study, we aimed to assess the safety of strain PS128^TM^ for use in humans via examining genotoxic and oral toxic effects using in vitro and in vivo testing. Five strains of *Salmonella typhimurium* were evaluated by the Ames test; no signs of increased reverse mutation were observed following exposure to PS128^TM^. Additional testing of Chinese hamster ovary (CHO) cells exposed to PS128^TM^ revealed that the incidence of chromosomal aberrations in CHO cells had not increased. PS128^TM^ treatment also did not affect the proportion of immature to total erythrocytes or the number of micronuclei in the immature erythrocytes of ICR mice. Moreover, following a 28 day study involving repeated oral dose toxicity tests (2400, 400, and 40 mg/kg body weight) utilizing an ICR mouse model, no observable adverse level (NOAEL) was found at any of the doses. PS128^TM^ was sensitive to antibiotics; however, genes related to the production of biogenic amines were absent. While further research is required, these toxicological assessments suggest that PS128^TM^ could be safe for human consumption.

## 1. Introduction

Probiotics are defined as live microorganisms that, when administered in adequate amounts, exert beneficial health effects on the host [1]. Numerous studies have demonstrated the diverse benefits of probiotics [2], including anti-inflammatory effects [3], gut protection [4], attenuation of metabolic dysfunction [5], regulation of immune response [6], anti-allergic effect [7], alleviation of diarrhea [8], beneficial alterations in intestinal flora, and alleviation of gastrointestinal motility disorders.

*Lactobacillus plantarum* PS128^TM^ was isolated from spontaneously fermented mustard greens in Taiwan [9]. Daily intake of this bacteria increased locomotor activity and dopamine and serotonin levels and reduced anxiety-like behavior in germ-free mice [9]. Serum analysis results showed reduced levels of corticosterone and inflammatory cytokines as well as increased levels of anti-inflammatory cytokines. Prefrontal cortex analysis revealed that dopamine levels were increased in early-life stressed mice [10]. In naïve mice, locomotor activity was increased, and anxiety-like behavior was decreased upon PS128^TM^ treatment. Prefrontal cortex analysis showed increased levels of dopamine and serotonin [11], suggesting that daily intake of PS128^TM^ exerts psychotropic effects that could reduce anxiety-like behavior and may be helpful in alleviating neuropsychiatric disorders by decreasing stress-related symptoms. Recently, evidence also showed that there are potentials for PS128^TM^ to improve cerebral dopamine-related symptoms in mice such as Tourette syndrome [12]. Although probiotics are extensively available to consumers and are commonly purchased as food probiotics (yogurts, cheeses, milk-based beverages, fermented fish, meats, and vegetables) or as food supplement probiotics (tablets, capsules, pills, powders, liquid concentrates in vials, and soft gels), the use of probiotics may not always be safe, and there are few reports on the side effects of probiotics, including those of *Lactobacillus* [13]. Thus, the present study was designed to examine the genotoxicity and subacute toxicity of *L plantarum* strain PS128^TM^.

## 2. Materials and Methods

### 2.1. PS128^TM^ Preparation

PS128^TM^ powder was provided by Bened Biomedical Co., Ltd. (Taipei, Taiwan). This bacterium was isolated from fermented mustard greens, which is a traditional Hakka ethnic food product. This novel strain was identified by phylogenetic classification of the 16S rDNA sequence. The isolated PS128^TM^ was inoculated into Man, Rogosa, and Sharpe broth (MRS) from BD Difco (Becton Dickinson, Franklin Lakes, NJ, USA), cultured at 37 °C for 18 h and then harvested by centrifugation at 6000× *g* for 10 min. The pellet was re-suspended in MRS plus 12.5% glycerol to a final concentration of 5 × 10^9^ colony-forming units (CFU) per milliliter. The re-suspended solution was then aliquoted in freezer tubes and stored at −20 °C until use. Before use, the aliquot was pre-warmed to 37 °C for 1 h and resuspended in saline. PS128^TM^ contains 100 billion CFU *L. plantarum* PS128^TM^ in 1 g powder (1 × 10^11^ CFU/g).

### 2.2. The Ames Salmonella/Microsome Mutagenicity Assay

Five strains of histidine-deficient *Salmonella typhimurium*—TA-102, TA-97, TA-98, TA-1535, and TA-100--were obtained from MolTox Inc. (Boone, NC, USA). The plate incorporation test performed both in the presence (0.5 mL S9 mix) and in the absence (0.5 mL of 0.2 M phosphate buffer, pH 7.4) of metabolic activation was previously described [14]. Briefly, 0.1 mL of an overnight bacterial culture (10^9^ cells/mL), 2 mL soft agar (5 mM histidine, 0.5% NaCl, 0.6% agar and 50 mM D-Biotin solution, 40–50 °C, pH around 7.4), 0.5 mL S9 mixture (if necessary), and five concentrations of PS128^TM^ ranging from 0.3125-5 mg/plate were combined in a test tube separately. After mixing, the sample was then poured into minimal glucose agar plates (Vogel-Bonner E medium containing 2% glucose with 1.5% agar). After incubating for 48 h at 37 °C in the dark, the number of his^+^ revertants for *Salmonella typhimurium* strains were counted macroscopically and compared to the negative control. A sample was considered positive when (1) a *p*-value less than 0.05 was found, (2) the number of revertants was at least double the spontaneous yield, and (3) a reproducible positive dose-response was present.

### 2.3. Cell Viability Assay

Chinese Hamster Ovary (CHO) K1 cell line purchased from the American Type Culture Collection (ATCC) was seeded into plastic 96-well plates and treated with different concentration of PS128^TM^ as described previously [15]. After treatment of 24 h, the media was removed and replaced with media containing 0.5 mg/mL of 3-(4,5-dimethylthiazol-2-yl)-2,5-diphenyltetrazolium bromide (MTT) solution to each well for another 1 h. Formazan crystals were dissolved by dimethyl sulfoxide, and the absorbance was measured by ELISA reader at 590 nm. Cell viability was normalized relative to the respective untreated control.

### 2.4. Chromosome Aberration Test

To investigate the clastogenic effects of PS128^TM^, chromatid breaks and polyploidy in metaphases were analyzed in CHO epithelial cells. Thus, 2 × 10^5^ cells/mL of cells were seeded in 6-cm dishes. PS128^TM^ powder was dissolved in distilled water before treatment and used at concentrations of 0.3125, 0.625, or 1.25 mg/mL. Mitomycin C (1 μg/mL) and benzo(a)pyrene (5 μg/mL) were added as positive control without or with S9 metabolic activation, respectively. After treatments, cells were exposed to Colcemid (0.1 μg/mL final concentration) for 2 h of the incubation period. The metaphase cells were then harvested by trypsinization and fixed with Carnoy’s solution (methanol: acetic acid, 3:1). The incidences of chromosomal aberrations from 100 metaphases per treatment were collected and scored. Data were shown as the average number of chromosomal aberrations per treatment ± standard deviation (SD) from 300 cells scored in three independent experiments.

### 2.5. In Vivo Micronucleus Assay

Twenty-five male ICR mice (8 weeks, National Laboratory Animal Center, Taipei, Taiwan) were acclimatized for 7 days and then randomly assigned to five cages for in vivo micronucleus assay. PS128^TM^ at doses of 0.5, 1, and 2 g/kg body weight were given by oral gavage in 5 male ICR mice once. Cyclophosphamide at 200 mg/kg body weight was intraperitoneal-injected as the positive control. At 24 h, 48 h, and 72 h, 10 μL blood samples from the tail vein were carefully collected onto a slide pre-coated with 40 μg/mL acridine orange. The incidence of micronucleated polychromatic erythrocytes (MNPCEs; ‰) and polychromatic erythrocytes (PCEs; %) to total erythrocytes were measured by calculating a total of 1000 erythrocytes or PCEs per animal by fluorescence microscopy, respectively.

### 2.6. 28 Day Subacute Toxicity Study

Forty male and forty female ICR mice (8 weeks, National Laboratory Animal Center, Taipei, Taiwan) were randomly allocated to cages (4 mice/cage) and underwent the 28 day subacute toxicity study treated with PS128^TM^ by oral gavage at doses of 0 (control), 40, 400, and 2400 mg/kg body weight. Body weight was measured twice a week. Water and food consumption were measured at 2 and 4 weeks. On day 29, all mice were sacrificed. Cage-side observations were conducted daily and included eyes, respiration, fur, motion, and the activity of all animals.

### 2.7. Serum Biochemical and Hematological Analysis

Animals were fasted overnight (16–18 h) and placed in an induction chamber with 2% isoflurane in 100% oxygen at a constant flow of 1.0 L/min using a Matrx VIP 3000 vaporizer (Midmark, Dayton, OH, USA) for general anesthesia at the end of the 28 day toxicity test. Blood samples from the abdominal aorta were collected for serum chemistry and hematology using an automatic analyzer. Clinical biochemistry analysis was performed using Fuji Dri-chem 4000i (Fujifilm Co., Tokyo, Japan), and sysmex XT-1800iv (TOA Medical Electronics Co., LtD., Kobe, Japan) was utilized for complete blood count and differential count (CBC/DC) analysis from whole blood.

### 2.8. Gross Necropsy

Gross necropsy was performed for analyzing external and internal features macroscopically [16,17]. After being carefully removed, extra fats around all vital organs were carefully removed. They were weighed separately followed by fixing in neutral-buffered formalin (10%). Absolute and relative terms were both expressed of organ wet weights. The fixed tissue segments were embedded in paraffin and stained with hematoxylin and eosin (H&E; Sigma-Aldrich, St. Louis, MO, USA) for histological assessment under a light microscope (Leica DM750; Leica Microsystems, Heerbrugg, Switzerland). After the mice were sacrificed, heart, lung, liver, spleen, kidney, adrenal gland, and gonads were carefully collected, and then the organ weights were measured. The ratio of organ weights was calculated as given below: ratio of organ weight (%) = organ weight (g)/body weight (g) × 100.

### 2.9. Histopathology

The biopsy sections of heart, lung, liver, spleen, kidney, adrenal gland, and gonads were fixed with 4% formaldehyde in phosphate-buffered saline (PBS) at pH 7.4. The fixed tissue segments were embedded in paraffin and stained with H&E (Sigma-Aldrich, St. Louis, MO, USA) for histological assessment under a light microscope (Leica DM750; Leica Microsystems, Heerbrugg, Switzerland).

### 2.10. Ethics Statement

All the animals were kept under a 12:12 h light/dark cycle (lights on: 20.00, lights off: 8.00) with ad libitum access to regular chow and water. Room temperature was maintained at 22.0 ± 2.0 °C with a relative humidity between 39 and 43%. The mice were maintained under a 12 h light/dark cycle at 22 ± 2 °C and 39–43% relative humidity. All animal handling procedures were reviewed and approved by the Institutional Animal Care and Use Committee (IACUC) of National Yang-Ming University (approval number: 1041112 and 1060606). All surgeries were executed under isoflurane anesthesia to minimize suffering. Animal testing criteria mandated that a loss over 20% of body weight related to the original weight was considered a humane endpoint in this study. None of the tested mice reached this humane endpoint during our study.

### 2.11. Antibiotic Resistance Profile

The antibiotic resistance profile for PS128^TM^ was evaluated according to the standard method procedure ISO 10932 (ISO 10932, 2010). The minimum inhibitory concentration (MIC) was determined after 48 h growth at 28 °C in an anaerobic chamber (Coy Laboratory Products, Grass Lake, MI, USA; atmosphere 85% N_2_, 5% CO_2_, 10% H_2_) for the following nine antimicrobial agents as recommended by European Food Safety Authority (EFSA) (EFSA, 2012). For each agent, the concentration range was expressed in μg/mL (given in brackets): gentamicin (1–256); kanamycin (2–256); streptomycin (4–256); tetracycline (0.5–128); erythromycin (0.0625–16); clindamycin (0.0625–16); chloramphenicol (0.5–32); ampicillin (0.25–32); and vancomycin (0.5–16). *L. plantarum* ATCC 14917T was used as the reference strain for quality control purposes (ISO 10932, 2010).

### 2.12. Detection of Amino Acid Decarboxylase Genes

Genes related to the production of biogenic amines, including *histidine decarboxylase* (*hdc*), *tyrosine decarboxylase* (tdc), and *ornithine decarboxylase* (*odc*), were detected by Polymerase chain reaction (PCR). Briefly, a 25 μL amplification reaction mixture containing 10 ng of template DNA, 20 mM Tris-HCl (pH 8.0), 50 mM KCl, 2.5 mM MgCl_2_, 200 μM of each deoxynucleoside triphosphate, 1 μM of each primer, and 1 U of AmpliTaq Gold DNA polymerase was performed in a PCR cycler (Gradient TurboCycler2, BlueRay BioTech, Taipei, Taiwan) using the following cycling parameters: 10 min for enzyme activation at 95 °C followed by 35 cycles of 60 s at 95 °C, 90 s at 53 °C, and 75 s at 72 °C, and a final extension step of 10 min at 72 °C. PCR products were resolved in 1% agarose gels and stained with ethidium bromide.

### 2.13. Statistical Analysis

Data are expressed as means ± SD. The differences between means were tested for statistical significance using a one-way ANOVA followed by a Dunnett’s multiple comparison test. Differences between the control group and other groups were considered statistically significant when *p* < 0.05 (*).

## 3. Results and Discussion

### 3.1. PS128^TM^ Treatment Was Negative to Mutagenicity and Clastogenicity Evaluation

PS128^TM^ at doses of 0.3125, 0.625, 1.25, 2.5, and 5 mg/plate both in the presence (0.5 mL S9 mix) and in the absence (0.5 mL of 0.2 M phosphate buffer, pH 7.4) of metabolic activation were tested for gene mutation using *Salmonella typhimurium* strains TA-97, TA-98, TA-102, TA-100, and TA1535. Results showed that the number of revertants was not elevated, while a significantly increased number of revertants was observed in positive controls (Table 1). In the MTT assay, CHO-K1 cells were treated with PS128^TM^ (0, 0.3125, 0.625, 1.25, 2.5, and 5 mg/mL) for 24 h, and the cell viability was 100 ± 0.0, 74.1 ± 15.6, 58.0 ± 5.4, 52.1 ± 8.8, 35.6 ± 10.1, and 26.9 ± 3.6, respectively, indicating the insoluble precipitation of PS128^TM^ may result in growth inhibition of CHO-K1 cells.

Therefore, PS128^TM^ at a dose of 1.25 mg/mL demonstrated cell viability of 52.1 ± 8.8 following treatment for 24 h and was used as the highest concentration for the chromosome aberration test. Results showed that treatment of PS128^TM^ did not increase the number of abnormal chromosomes (Table 2). Also, the percentage of reticulocytes to the total erythrocytes (%) and the incidence of MNPCEs in ICR mice treated with PS128^TM^ at 0.5, 1, and 2 g/kg body weight indicated no significant difference compared to the control group (distilled water) by in vivo micronucleus assay (Table 3). All together, these results showed that PS128^TM^ was negative to mutagenicity and clastogenicity evaluation.

### 3.2. PS128^TM^ did not Induce Adverse Effects in the 28 Day Subacute Toxicity Test

Following 28 days of repeated oral administration of *L. plantarum* PS128^TM^ powder, no abnormal clinical signs and/or mortality were detected in the treatment group (0, 40, 400, and 2400 mg/kg body weight). Although the body weight of each group increased, it was not significant (Table 4). Food and water consumption in low-, medium-, and high-dose groups in each week showed no significant difference compared with the control group (data not shown). After 28 days of repeated oral administration of *L. plantarum* PS128^TM^ powder, whole blood was collected and the components analyzed, and the results are depicted in Table 5. Significant differences were observed in mean corpuscular hemoglobin (MCH) and mean corpuscular volume (MCV) in the male groups and in white cell blood count (WBC) and neutrophils in the female groups; however, these differences were not dose-dependent, and all the values were in the normal range [18]. Biochemical analyses were performed using whole blood to analyze various blood components, and the results are depicted in Table 6. In male mice, the glucose value in low-, medium-, and high-dose groups was lower than that of the control group (*p* < 0.05). In female mice, the aspartate aminotransferase (AST) values for low-, medium-, and high-dose groups were found to be lower than the control group (*p* < 0.05). The lactate dehydrogenase (LDH) value in low- and high-dose groups was lower than the control group (*p* < 0.05). The triglycerides value in the high-dose group was higher than the control group (*p* < 0.05). The Ca value in the high-dose group was higher than that of the control group (*p* < 0.05). These changes were not dose-dependent, and the values were in the normal range [18]; therefore, they were not considered toxic responses.

### 3.3. PS128^TM^ did not Cause Macropathological or Histopathological Lesions in the 28 Day Subacute Toxicity Test

**Figure 1:**
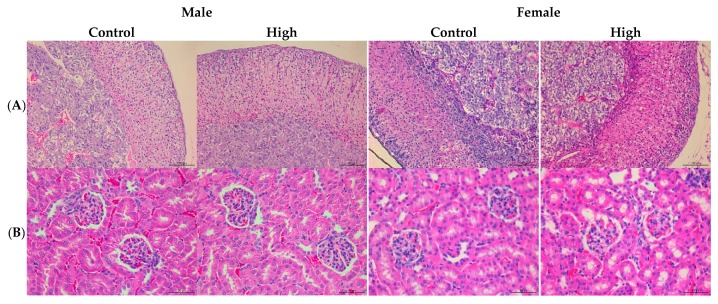

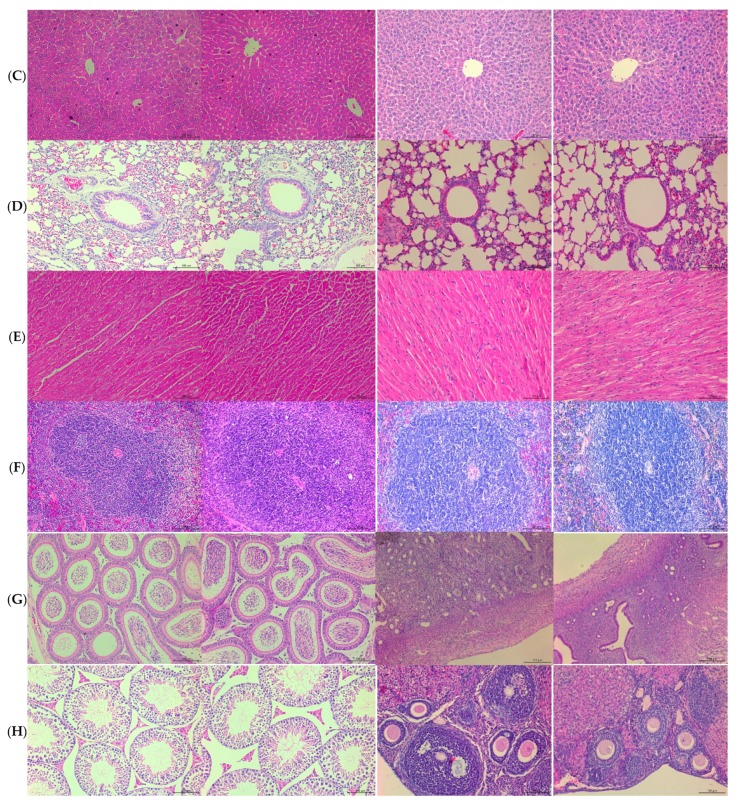
Histopathological changes in the ICR mouse model during the PS128^TM^ 28 day toxicity test. Representative histopathological pictures from the male control ICR mice and high-dose (2400 mg/kg) groups of adrenal (**A**), kidney (**B**), liver (**C**), lung (**D**), heart (**E**), spleen (**F**), epididymis (**G**), and testis (**H**) (H&E stain, 200×) are depicted in the left panel; Representative histopathological pictures from the female control ICR mice and high-dose (2400 mg/kg) group of adrenal (**A**), kidney (**B**), liver (**C**), lung (**D**), heart (**E**), spleen (**F**), uterus (**G**), and ovary (**H**) (H&E stain, 200×) are depicted in the right panel.

After the mice were euthanized, heart, lung, liver, spleen, kidney, adrenal gland, and gonads (male: epididymis and testis; female: ovary and uterus) were carefully collected, and the organ weights were measured. The results are shown in Table 7. The organ weight and the ratio of organ weight showed no significant differences compared with the control group (*p* < 0.05). Histological assessment of heart, lung, liver, spleen, kidney, adrenal gland, and gonads (male: epididymis and testis; female: ovary and uterus) was performed to estimate the effect of 28 day repeated oral administration of *L. plantarum* PS128^TM^. Low-, medium-, and high-dose groups showed no significant differences in either male or female mice when compared with the control group. Histopathological photographs of the control and the high-dose (2400 mg/kg) group were presented in Figure 1.

### 3.4. PS128^TM^ Was Sensitive to Antibiotics

PS128^TM^ was challenged with nine antimicrobial agents and grown for 48 h growth at 28 °C in an anaerobic chamber, and then the MIC was determined for each agent. MIC values for antibiotic susceptibility of PS128^TM^ against the tested antibiotics are depicted in Table 8. PS128^TM^ exhibited MIC values lower than the MIC breakpoint values recommended for heterofermentative *Lactobacillus* strains by EFSA for all the tested antibiotics (ampicillin, gentamicin, kanamycin, erythromycin, clindamycin, streptomycin, tetracycline, vancomycin, and chloramphenicol). Since PS128^TM^ was not found to be resistant to the tested antibiotics, no further studies on their antibiotic resistance are required according to EFSA.

### 3.5. No Genes Related to the Production of Biogenic Amines Were Found in PS128^TM^

Some *Lactobacilli* are capable of producing biogenic amines [19], which are generally considered undesirable metabolites since the alimentary intake of biogenic amines at higher concentrations results in adverse effects to the health of consumers [20]. Thus, to investigate if *L. plantarum* PS128^TM^ harbors genes related to the production of biogenic amines (*hdc*, *tdc*, and *odc* genes), PCR-based detections were performed, and the results showed the absence of these genes in the genome of *L. plantarum* PS128^TM^ (Table 9).

## 4. Conclusions

The results of the Ames *Salmonella*/microsome mutagenicity assay, the chromosome aberration test, and the mammalian in vivo micronucleus assay showed that the probiotic PS128^TM^ isolated from fermented mustard greens did not exert a genotoxic effect. The NOAEL of PS128^TM^ was set to be greater than 2.4 g/kg body weight for both male and female ICR mice. PS128^TM^ was sensitive to antibiotics, and no genes related to the production of biogenic amines were detected. These results were summarized and indicated the safety of PS128^TM^ for human consumption (Table 10).

## Figures and Tables

**Table 1:** The Ames *Salmonella*/microsome mutagenicity assay of PS128.

	TA97	TA98	TA100	TA102	TA1535
**Without S9 Metabolic Activation (Number/Plate)**					
Negative ^1^	37 ± 6	15 ± 7	129 ± 15	7 ± 0	17 ± 3
Positive ^2^	467 ± 64 *	2208 ± 200 *	1249 ± 76 *	413 ± 21 *	227 ± 7 *
**PS128 (mg/Plate)**					
0.3125	26 ± 11	18 ± 4	167 ± 2	5 ± 2	11 ± 2
0.625	34 ± 8	18 ± 1	177 ± 6	4 ± 1	17 ± 2
1.25	36 ± 5	19 ± 4	170 ± 1	8 ± 2	14 ± 2
2.5	35 ± 5	20 ± 2	147 ± 11	11 ± 7	12 ± 2
5	21 ± 4	15 ± 2	120 ± 7	6 ± 1	12 ± 5
**With S9 Metabolic Activation (Number/Plate)**					
Negative ^1^	100 ± 13	24 ± 8	37 ± 7	17 ± 1	18 ± 13
Positive ^2^	1220 ± 73 *	296 ± 7 *	2004 ± 178 *	518 ± 19 *	168 ± 29 *
**PS128 (mg/Plate)**					
0.3125	93 ± 12	29 ± 3	33 ± 4	16 ± 1	10 ± 1
0.625	82 ± 2	25 ± 4	34 ± 5	17 ± 4	8 ± 3
1.25	85 ± 6	28 ± 5	33 ± 5	20 ± 4	15 ± 9
2.5	79 ± 14	31 ± 2	40 ± 4	17 ± 4	11 ± 1
5	95 ± 25	18 ± 4	34 ± 5	16 ± 1	14 ± 5

Data are expressed as mean ± SD (*N* ≥ 3). ^1^ Solvent (distilled water) was used as negative control. ^2^ Positive control in plate without s9 metabolic activation: 9-Aminoacridine 1 μg/plate (TA97); 4-Nitro-o-phenylenediamine 1 μg/plate (TA98, TA100 and TA102); Sodium Azide 5 μg/plate (TA1535); 2-aminoanthracene (10 μg/plate) for all strains with s9 metabolic activation. * was labeled when the number of revertants was twice that of negative control in TA98, TA100, and TA102 or triple that of negative control in TA97 andTA1535.

**Table 2:** Chromosome aberration test of PS128 in Chinese Hamster Ovary (CHO) cells.

	Aberrant Cell (%) ^4^		Number of Cells with Structural Aberrations (%) ^3^					
	With Gap	Without Gap	G	B	D	g	b	e
	**3 h without S9 Metabolic Activation**							
Negative ^1^	0.3 ± 0.5	0.0 ± 0.0	0.0 ± 0.0	0.0 ± 0.0	0.0 ± 0.0	0.3 ± 0.5	0.0 ± 0.0	0.0 ± 0.0
Positive ^2^	7.5 ± 1.9 ***	5.3 ± 1.0 ***	1.3 ± 0.5 ***	0.8 ± 0.5 **	0.8 ± 1.0	1.0 ± 0.8	1.0 ± 0.8 *	1.3 ± 1.0 **
**PS128 (mg/mL)**								
0.3125	0.0 ± 0.0	0.0 ± 0.0	0.0 ± 0.0	0.0 ± 0.0	0.0 ± 0.0	0.0 ± 0.0	0.0 ± 0.0	0.0 ± 0.0
0.625	0.3 ± 0.5	0.0 ± 0.0	0.0 ± 0.0	0.0 ± 0.0	0.0 ± 0.0	0.3 ± 0.5	0.0 ± 0.0	0.0 ± 0.0
1.25	0.3 ± 0.5	0.3 ± 0.5	0.0 ± 0.0	0.0 ± 0.0	0.0 ± 0.0	0.0 ± 0.0	0.3 ± 0.5	0.0 ± 0.0
**3 h with S9 Metabolic Activation**								
Negative ^1^	0.5 ± 0.6	0.0 ± 0.0	0.0 ± 0.0	0.0 ± 0.0	0.0 ± 0.0	0.5 ± 0.6	0.0 ± 0.0	0.0 ± 0.0
Positive ^2^	7.5 ± 1.3 ***	5.5 ± 1.3 ***	1.3 ± 1.0 **	0.3 ± 0.5	1.3 ± 1.0 **	0.8 ± 0.5	1.3 ± 1.0 *	1.5 ± 1.0 **
**PS128 (mg/mL)**								
0.3125	0.0 ± 0.0	0.0 ± 0.0	0.0 ± 0.0	0.0 ± 0.0	0.0 ± 0.0	0.0 ± 0.0	0.0 ± 0.0	0.0 ± 0.0
0.625	0.5 ± 1.0	0.3 ± 0.5	0.0 ± 0.0	0.0 ± 0.0	0.0 ± 0.0	0.3 ± 0.5	0.3 ± 0.5	0.0 ± 0.0
1.25	0.0 ± 0.0	0.0 ± 0.0	0.0 ± 0.0	0.0 ± 0.0	0.0 ± 0.0	0.0 ± 0.0	0.0 ± 0.0	0.0 ± 0.0
**24 h without S9 Metabolic Activation**								
Negative ^1^	0.8 ± 1.0	0.3 ± 0.5	0.0 ± 0.0	0.0 ± 0.0	0.0 ± 0.0	0.5 ± 1.0	0.3 ± 0.5	0.0 ± 0.0
Positive ^2^	8.0 ± 2.2 ***	5.8 ± 1.7 ***	1.3 ± 0.5 ***	0.5 ± 0.6	1.5 ± 1.3 *	1.0 ± 0.8	1.0 ± 0.8	2.0 ± 0.8 ***
**PS128 (mg/mL)**								
0.3125	0.3 ± 0.5	0.3 ± 0.5	0.0 ± 0.0	0.0 ± 0.0	0.0 ± 0.0	0.0 ± 0.0	0.3 ± 0.5	0.0 ± 0.0
0.625	0.3 ± 0.5	0.0 ± 0.0	0.0 ± 0.0	0.0 ± 0.0	0.0 ± 0.0	0.3 ± 0.5	0.0 ± 0.0	0.0 ± 0.0
1.25	0.3 ± 0.5	0.0 ± 0.0	0.0 ± 0.0	0.0 ± 0.0	0.0 ± 0.0	0.3 ± 0.5	0.0 ± 0.0	0.0 ± 0.0

Data are shown as the mean ± SD. (*N* = 3). * *p* < 0.05, ** *p* < 0.01, *** *p* < 0.001 indicates significant difference compared with control group by one-way Anova. ^1^ Distilled water was used as negative control. ^2^ Mitomycin C 1 μg/mL was used as positive control without S9 metabolic activation condition; benzo(a)pyrene 5 μg/mL was used as positive control with S9 metabolic activation condition. ^3^ G: chromosome gap; B: chromosome break; D: dicentric; g: chromatid gap; b: chromatid break; e: exchange. ^4^ Chromatid and chromosome gaps were recorded but separated into two groups, with gaps and without gaps.

**Table 3:** In vivo micronucleus assay of PS128 in male ICR mice.

	Negative	PS128 (g/kg Body Weight)			Positive
	Distilled Water	0.5	1.0	2.0	Cyclophosphamide 200 mg/kg
**Number of Micronuclei (%)**					
Day 1	0.4 ± 0.5	0.4 ± 0.5	0.2 ± 0.4	0.4 ± 0.5	2.8 ± 0.4 ***
Day 2	0.4 ± 0.5	0.2 ± 0.4	0.6 ± 0.5	0.6 ± 0.5	3.4 ± 0.5 ***
Day 3	0.6 ± 0.5	0.4 ± 0.5	0.6 ± 0.5	0.4 ± 0.5	3.2 ± 0.8 ***
**Reticuloctes (%)**					
Day 1	5.2 ± 0.8	5.4 ± 0.5	4.8 ± 0.8	5.6 ± 0.5	3.4 ± 0.5 ***
Day 2	5.2 ± 1.3	5.4 ± 0.9	6.0 ± 1.0	5.2 ± 1.1	2.8 ± 0.8 ***
Day 3	5.8 ± 1.1	5.2 ± 1.3	5.6 ± 1.3	5.2 ± 1.3	3.6 ± 1.1 ***

Data are shown as the Mean ± SD. (*N* = 5). *** *p* < 0.001 indicates significant difference compared with control group by one-way Anova.

**Table 4:** Body weight changes of male and female ICR mice during the 28 day toxicity test.

**Gender**	**Male**			
**PS128 (mg/kg (Body Weight/Day))**	**0 mg/kg Control**	**40 mg/kg Low**	**400 mg/kg Middle**	**2400 mg/kg High**
**Number of Animals**	**10**	**10**	**10**	**10**
	**Mean of Body Weight per Day in Different Week (g)**			
Week 0	33.3 ± 1.5	34.6 ± 1.3	34.5 ± 1.9	34.4 ± 1.8
Week 1	35.0 ± 2.2	35.4 ± 1.2	34.7 ± 1.6	34.4 ± 2.0
Week 2	36.1 ± 2.4	35.8 ± 1.4	35.3 ± 1.6	34.8 ± 2.1
Week 3	36.4 ± 2.4	35.9 ± 1.8	35.9 ± 2.0	35.9 ± 3.0
Week 4	36.3 ± 2.3	36.2 ± 1.8	36.3 ± 2.2	36.6 ± 3.2
**Gender**	**Female**			
**PS128 (mg/kg (Body Weight/Day))**	**0 mg/kg Control**	**40 mg/kg Low**	**400 mg/kg Middle**	**2400 mg/kg High**
**Number of Animals**	**10**	**10**	**10**	**10**
	**Mean of Body Weight per Day in Different Week (g)**			
Week 0	26.1 ± 1.4	27.2 ± 1.8	27.1 ± 1.3	27.6 ± 1.7
Week 1	26.3 ± 1.5	27.3 ± 2.0	27.2 ± 1.0	27.3 ± 1.2
Week 2	27.3 ± 1.5	27.8 ± 1.9	28.1 ± 1.5	28.2 ± 1.3
Week 3	27.9 ± 1.3	29.4 ± 2.6	29.0 ± 1.7	29.1 ± 1.8
Week 4	28.6 ± 1.7	29.7 ± 2.0	30.4 ± 2.5	29.7 ± 1.8

**Table 5:** Complete blood count (CBC) analysis of subacute toxicity after 28 day PS128 treatment on ICR mice.

		Male				Female			
		0	40	400	2400	0	40	400	2400
Red blood cell count (RBC)	10^6^/dL	9.10 ± 1.32	9.82 ± 0.66	9.59 ± 0.22	8.84 ± 2.55	9.15 ± 0.32	9.30 ± 0.48	9.30 ± 0.53	8.67 ± 2.79
Hematocrit (HCT)	%	45.30 ± 6.10	46.68 ± 2.77	45.99 ± 1.78	42.53 ± 12.03	45.91 ± 1.64	46.06 ± 1.78	47.12 ± 2.14	46.02 ± 1.23
Hemoglobin (Hb)	g/L	145.69 ± 12.89	147.40 ± 9.73	146.00 ± 4.71	135.50 ± 41.05	144.10 ± 4.04	146.00 ± 6.48	149.10 ± 6.40	150.60 ± 6.90
Mean corpuscular hemoglobin (MCH)	Pg	16.01 ± 2.89	15.02 ± 0.50 *	15.22 ± 0.42 *	15.05 ± 1.24	15.75 ± 0.24	15.73 ± 0.76	16.06 ± 0.52	15.33 ± 1.75
MCH concentration (MCHC)	g/dL	31.40 ± 5.39	31.58 ± 1.29	31.75 ± 0.66	31.14 ± 2.93	31.41 ± 1.18	31.72 ± 1.03	31.66 ± 0.68	31.45 ± 4.08
Mean corpuscular volume (MCV)	fL	50.36 ±2.30	47.60 ± 1.70 *	47.96 ± 1.33 *	48.44 ± 2.26 *	50.25 ± 2.53	49.64 ± 2.37	50.76 ± 1.92	48.93 ±2.41
RBC distribution width coefficient of variation (RDW-CV)	%	19.39 ± 2.01	19.87 ± 1.10	19.33 ± 0.45	18.95 ± 2.06	19.24 ± 0.49	19.33 ± 0.86	19.13 ± 0.69	18.69 ± 2.15
RBC distribution width standard deviation (RDW-SD)	fL	31.39 ± 2.46	29.60 ± 2.16	29.28 ± 0.90	28.93 ± 2.13	30.72 ± 1.46	30.30 ± 1.44	30.80 ± 1.96	28.67 ± 2.45
Platelet distribution width (PDW)	fL	6.77 ± 0.32	7.15 ± 0.34	6.77 ± 0.24	6.75 ± 0.54	6.79 ± 0.35	6.89 ± 0.20	7.09 ± 0.52	6.84 ± 0.34
Mean platelet volume (MPV)	fL	6.62 ± 0.36	6.69 ± 0.27	6.41 ± 0.21	6.51 ± 0.15	6.59 ± 0.28	6.58 ± 0.21	6.63 ± 0.36	6.59 ± 0.24
White blood cell count (WBC)	10^3^/μL	5.74 ± 2.40	5.46 ± 2.38	5.13 ± 1.30	5.86 ± 3.09	3.38 ± 0.56	5.30 ± 1.59 *	5.29 ± 1.83 *	5.97 ± 2.26 *
Lymphocytes	%	82.91 ± 4.44	80.84 ± 8.29	85.87 ± 4.03	82.92 ± 3.07	79.90 ± 5.07	84.72 ± 5.86	85.37 ± 4.18	85.61 ± 6.32
Neutrophils	%	13.79 ± 4.31	15.53 ± 6.09	11.78 ± 3.23	14.07 ± 2.94	16.43 ± 3.09	12.38 ± 4.84	12.02 ± 4.37 *	11.44 ± 6.07 *
Monocytes	%	1.45 ± 0.55	1.03 ± 0.41	0.83 ± 0.42	1.10 ± 0.49	0.44 ± 0.20	1.04 ± 0.80	0.99 ± 0.43	0.96 ± 0.51
Eosinophil	%	1.54 ± 1.14	1.53 ± 0.69	1.38 ± 0.67	1.77 ± 1.31	1.25 ± 0.79	1.75 ± 0.70	1.57 ± 0.62	1.84 ± 1.74
Basophils	%	0.11 ± 0.12	0.08 ± 0.10	0.14 ± 0.13	0.14 ± 0.13	0.30 ± 0.48	0.11 ± 0.25	0.05 ± 0.11	0.14 ± 0.12
Platelets count (PLT)	10^6^/μL	1.23 ± 0.56	1.48 ± 0.41	1.59 ± 0.11	1.39 ± 0.49	1.29 ± 0.30	1.41 ± 0.22	1.37 ± 0.26	1.31 ± 0.30
Platelet large cell ratio (P-LCR)	%	4.94 ± 1.67	5.24 ± 1.61	3.84 ± 0.87	4.35 ± 1.03	4.46 ± 1.68	4.53 ± 1.08	4.68 ± 1.72	4.33 ± 1.09
Plateletcrit (PCT)	%	0.81 ± 0.38	0.99 ± 0.27	1.02 ± 0.08	0.91 ± 0.32	0.85 ± 0.19	0.93 ± 0.15	0.91 ± 0.19	0.80 ± 0.30

Data are expressed as mean ± S.D. Significant difference between control treated group at * *p* < 0.05 by one-way ANOVA.

**Table 6:** Biochemical analysis of subacute toxicity after 28 day PS128 treatment on ICR mice.

		Male				Female			
Daily Dose (g/kg (b.w/day))		0	0.04	0.4	2.4	0	0.04	0.4	2.4
Calcium	mg/dL	7.88 ± 0.68	8.09 ± 1.08	8.35 ± 0.47	8.78 ± 0.58	8.30 ± 0.60	8.53 ± 0.55	8.59 ± 0.55	8.88 ± 0.73 *
Chloride	mmol/L	84.80 ± 1.30	84.30 ± 1.34	83.60 ± 2.12	85.40 ± 1.65	85.20 ± 2.74	84.40 ± 2.17	84.50 ± 1.90	84.50 ± 1.08
Phosphorus	mg/dL	6.50 ± 1.05	6.10 ± 1.02	6.01 ± 1.16	6.67 ± 0.75	6.79 ± 0.51	6.31 ± 0.74	6.44 ± 0.63	6.86 ± 0.97
Potassium	mmol/L	4.24 ± 0.57	4.01 ± 0.50	4.13 ± 0.36	4.40 ± 0.49	4.02 ± 0.30	4.39 ± 0.26	4.33 ± 0.32	4.17 ± 0.52
Sodium	mmol/L	130.40 ± 1.34	129.90 ± 1.37	129.90 ± 1.73	130.10 ± 0.74	132.60 ± 2.17	132.50 ± 2.99	132.40 ± 1.51	132.10 ± 2.08
Glucose	mg/dL	125.00 ± 61.53	75.40 ± 41.40 *	98.50 ± 28.27	87.10 ± 21.05 *	119.40 ± 22.32	140.60 ± 43.39	155.30 ± 36.95	150.20 ± 37.32
Total bilirubin (TBIL)	mg/dL	0.12 ± 0.04	0.24 ± 0.18	0.24 ± 0.11	0.20 ± 0.05	0.41 ± 0.11	0.37 ± 0.11	0.44 ± 0.13	0.41 ± 0.14
Alanine aminotransferase (ALT)	U/L	68.80 ± 60.14	66.40 ± 31.02	51.80 ± 37.95	49.50 ± 51.60	40.10 ± 18.02	27.90 ± 9.36	35.89 ± 13.17	30.10 ± 9.89
Aspartate aminotransferase (AST)	U/L	102.40 ± 22.21	87.50 ± 25.89	76.60 ± 17.71	76.60 ± 28.58	109.90 ± 27.31	76.80 ± 12.35 *	87.40 ± 18.81 *	82.00 ± 18.35 *
Alkaline phosphatase (ALP)	U/L	238.40 ± 89.90	210.50 ± 69.02	228.10 ± 51.36	265.70 ± 65.13	340.40 ± 55.86	354.80 ± 126.63	303.80 ± 67.18	365.40 ± 82.67
Creatinine	mg/dL	0.24 ± 0.05	0.24 ± 0.10	0.16 ± 0.08	0.20 ± 0.09	0.23 ± 0.05	0.19 ± 0.07	0.18 ± 0.04	0.19 ± 0.06
Blood urea nitrogen (BUN)	mg/dL	34.28 ± 3.44	35.86 ± 5.53	30.99 ± 1.66	32.04 ± 4.38	23.58 ± 4.03	26.61 ± 5.39	27.01 ± 7.29	29.69 ± 8.24
Albumin	g/dL	2.88 ± 0.23	3.00 ± 0.38	3.15 ± 0.24	3.23 ± 0.19	3.28 ± 0.52	3.34 ± 0.35	2.93 ± 1.04	3.15 ± 0.33
Total protein	g/dL	5.79 ± 0.42	5.93 ± 0.40	6.00 ± 0.33	6.30 ± 0.37	6.15 ± 0.73	6.37 ± 0.30	6.22 ± 0.33	6.09 ± 0.41
Cholesterol	mg/dL	109.40 ± 17.80	126.40 ± 16.29	128.80 ± 31.00	140.25 ± 22.48	104.00 ± 14.16	111.70 ± 16.80	97.90 ± 22.11	119.20 ± 24.54
Triglycerides	mg/dL	106.40 ± 15.19	96.90 ± 23.52	128.70 ± 35.81	143.80 ± 47.87	86.70 ± 27.54	118.10 ± 52.60	123.00 ± 33.11 *	78.30 ± 31.70
Lactate dehydrogenase (LDH)	U/L	499.40 ± 246.70	525.00 ± 274.47	470.40 ± 284.01	355.70 ± 223.76	276.33 ± 28.74	293.30 ± 96.82	334.70 ± 104.52	303.60 ± 87.05
Amylase	U/L	1034.20 ± 95.16	973.70 ± 124.57	882.20 ± 272.25	1119.00 ± 222.90	885.00 ± 152.56	1082.20 ± 289.52	857.30 ± 128.63	819.10 ± 196.25

Data are expressed as mean ± S.D. Significant difference between control treated group at * *p* < 0.05 by one-way ANOVA.

**Table 7:** Organ weight of subacute toxicity after 28 day PS128 treatment.

		Male				Female			
Daily Dose (mg/kg)		0	40	400	2400	0	40	400	2400
Adrenals									
Absolute Weight	mg	5.09 ± 1.15	4.89 ± 0.89	4.81 ± 1.20	4.33 ± 0.71	14.17 ± 8.53	7.89 ± 2.05	9.78 ± 2.74	9.42 ± 2.47
Ratio per Body Weight	(10^−3^)	0.01 ± 0.00	0.01 ± 0.00	0.01 ± 0.00	0.01 ± 0.00	0.05 ± 0.03	0.03 ± 0.01	0.03 ± 0.01	0.03 ± 0.01
Heart									
Absolute Weight	mg	153.00 ± 9.49	162.00 ± 20.98	146.00 ± 11.74	140.00 ± 18.86	121.00 ± 11.01	133.00 ± 15.67	132.00 ± 11.35	129.00 ± 14.49
Ratio per Body Weight	(10^−3^)	0.41 ± 0.04	0.45 ± 0.06	0.40 ± 0.04	0.38 ± 0.05	0.42 ± 0.03	0.45 ± 0.05	0.43 ± 0.03	0.43 ± 0.05
Kidneys									
Absolute Weight	mg	517.00 ± 41.11	514.00 ± 60.22	488.00 ± 59.78	477.00 ± 46.20	365.00 ± 25.50	377.00 ± 35.61	385.00 ± 49.72	372.00 ± 23.48
Ratio per Body Weight	(10^−3^)	1.40 ± 0.09	1.42 ± 0.19	1.35 ± 0.13	1.31 ± 0.14	1.27 ± 0.11	1.27 ± 0.11	1.26 ± 0.12	1.25 ± 0.07
Liver									
Absolute Weight	mg	1703 ± 163.91	1509 ± 219.42	1423 ± 103.39	1443 ± 143.53	1200 ± 133.67	1240 ± 217.61	1354 ± 218.39	1279 ± 203.06
Ratio per Body Weight	(10^−3^)	4.59 ± 0.34	4.15 ± 0.45	3.94 ± 0.30	3.96 ± 0.45	4.17 ± 0.31	4.18 ± 0.75	4.41 ± 0.45	4.27 ± 0.44
Spleen									
Absolute Weight	mg	119.00 ± 32.81	106.00 ± 27.16	93.00 ± 22.14	78.00 ± 19.32	115.00 ± 21.21	95.00 ± 23.21	101.00 ± 18.53	104.00 ± 22.21
Ratio per Body Weight	(10^−3^)	0.32 ± 0.07	0.29 ± 0.06	0.26 ± 0.06	0.21 ± 0.05	0.40 ± 0.05	0.32 ± 0.08	0.33 ± 0.05	0.35 ± 0.06
Testis/Ovary									
Absolute Weight	mg	228.00 ± 22.01	217.00 ± 46.92	220.00 ± 35.28	222.00 ± 37.95	24.00 ± 5.16	22.00 ± 6.32	28.00 ± 7.89	27.00 ± 6.75
Ratio per Body Weight	(10^−3^)	0.62 ± 0.07	0.60 ± 0.14	0.61 ± 0.09	0.61 ± 0.13	0.08 ± 0.02	0.07 ± 0.02	0.09 ± 0.03	0.09 ± 0.02
Epididymis/Uterus									
Absolute Weight	mg	53.00 ± 9.49	54.00 ± 8.97	45.00 ± 5.27	50.00 ± 8.16	142.00 ± 72.23	153.00 ± 61.29	138.00 ± 56.53	125.00 ± 41.97
Ratio per Body Weight	(10^−3^)	0.14 ± 0.03	0.15 ± 0.06	0.12 ± 0.01	0.14 ± 0.03	0.49 ± 0.25	0.51 ± 0.19	0.45 ± 0.19	0.42 ± 0.13
Lung									
Absolute Weight	mg	195.00 ± 12.69	193.00 ± 18.29	183.00 ± 17.67	175.00 ± 17.16	176.00 ± 10.75	175.00 ± 8.50	176.00 ± 19.55	175.00 ± 14.34
Ratio per Body Weight	(10^−3^)	0.53 ± 0.05	0.53 ± 0.04	0.51 ± 0.05	0.48 ± 0.05	0.61 ± 0.05	0.59 ± 0.04	0.58 ± 0.04	0.59 ± 0.04

**Table 8:** *L. plantarum* PS128 susceptibility towards nine antibiotics.

Antibiotics	Cut-Off Values of *L. plantarum* ^a^ (mg/L)	PS128	
		MICs (mg/L)	Interpretation
ampicillin	2	<0.25	S
vancomycin	n.r.	n.r.	n.r.
gentamicin	16	2	S
kanamycin	64	32	S
streptomycin	n.r.	n.r.	n.r.
erythromycin	1	0.125	S
clindamycin	2	2	S
tetracycline	32	8	S
chloramphenicol	8	2	S

^a^ according to the guidance recommended by EFSA (2012); n.r., not required; S, sensitive; MIC, minimum inhibitory content.

**Table 9:** Presence and primer sequences utilized in the detection of genes related to the production of biogenic amines in *L. plantarum* PS128.

Genes	Presence of Genes	Primer Sequence (5’–3’)
*hdc*	-	AGATGGTATTGTTTCTTATG
		AGACCATACACCATAACCTT
*tdc*-1	-	GAYATNATNGGNATNGGNYTNGAYCARG
		CCRTARTCNGGNATAGCRAARTCNGTRTG
*tdc*-1	-	CCACTGCTGCATCTGTTTG
		CCRTARTCNGGNATAGCRAARTCNGTRTG
*odc*-2	-	GTNTTYAAYGCNGAYAARACNTAYTTYGT
		TACRCARAATACTCCNGGNGGRTANGG
*odc*-2	-	GTNTTYAAYGCNGAYAARCANTAYTTYGT
		ATNGARTTNAGTTCRCAYTTYTCNGG

**Table 10:** Summary of toxicity studies of *Lactobacillus plantarum* PS128TM.

Assay	Purpose	Reference	Results
The Ames *Salmonella*/microsome mutagenicity assay	To assess the potential mutagenic effect using the bacterial strain *Salmonella typhimurium*	[21]	Negative
Chromosome aberration test	To assess the potential mutagenic effect in vitro	[22]	Negative
Micronucleus assay	To assess the potential mutagenic effect in vivo	[23]	Negative
28 day subacute toxicity study	To exam the possible health hazards likely to arise from repeated exposure of PS128	[16]	Negative; NOAEL: more than 2.4 g/kg
Antibiotic resistance profile	To test the possibility of resistance to antibiotics	[24]	Negative
Detection of amino acid decarboxylase genes	To detect the possible biogenic amine-producing potential	[20]	Negative
